# Spliceosomal introns in the diplomonad parasite *Giardia duodenalis* revisited

**DOI:** 10.1099/mgen.0.001117

**Published:** 2023-11-07

**Authors:** Matthew H. Seabolt, Dawn M. Roellig, Konstantinos T. Konstantinidis

**Affiliations:** ^1^​ Division of Foodborne, Waterborne, and Environmental Diseases, National Center for Emerging and Zoonotic Infectious Diseases, Centers for Disease Control and Prevention, Atlanta, GA 30329, USA; ^2^​ School of Biological Sciences, Georgia Institute of Technology, Atlanta, GA 30332, USA; ^3^​ Leidos Inc., Reston, VA 20190, USA; ^4^​ School of Civil and Environmental Engineering, Georgia Institute of Technology, Atlanta, GA 30332, USA

**Keywords:** gene expression, genome annotation, *Giardia duodenalis*, introns, splicing

## Abstract

Complete reference genomes, including correct feature annotations, are a fundamental aspect of genomic biology. In the case of protozoan species such as *Giardia duodenalis*, a major human and animal parasite worldwide, accurate genome annotation can deepen our understanding of the evolution of parasitism and pathogenicity by identifying genes underlying key traits and clinically relevant cellular mechanisms, and by extension, the development of improved prevention strategies and treatments. This study used bioinformatics analyses of *Giardia* mRNA libraries to characterize known introns and identify new intron candidates, working towards completion of the *G. duodenalis* assemblage A strain ‘WB’ genome and further elucidating *Giardia*’s gene expression. By using a set of experimentally validated positive control loci to calibrate our intron detection pipeline, we were able to detect evidence of previously missed candidate splice junctions directly from expressed transcript data. These intron candidates were further studied *in silico* using NMDS (non-metric multidimensional scaling) clustering to determine shared characteristics and their relative importance such as secondary structure, splicing efficiency and motif conservation, and thus to refine intron models. Results from this study identified 34 new intron candidates, with several potential introns showing evidence that secondary structure of the mRNA molecule might play a more significant role in splicing than previously reported eukaryotic splicing activity mediated by a reduced spliceosome present in *G. duodenalis*.

## Data Summary

This study did not generate new sequencing data. All data used for bioinformatic analysis in this study are publicly available and were collected from NCBI’s GEO and SRA databases from accession numbers provided in the Methods. Updated feature annotation files have been submitted to GiardiaDB (giardiadb.org) for review.

### Impact Statement

*Giardia duodenalis* is a globally ubiquitous parasite of humans and wildlife that causes hundreds of millions of infections per year and has a high propensity to cause large waterborne disease outbreaks. Despite its public health importance, relatively few genomes of *Giardia* have been sequenced to date and a reference genome for this organism remains incomplete nearly 15 years after initial publication. This study focuses on feature annotation of the most complete *Giardia* genome, *G. duodenalis* assemblage A ‘WB’ strain, utilizing strand-specific mRNA transcriptome sequences and eight previously characterized *cis*-splicing introns acting as positive controls to examine splicing activity in *Giardia*. This work is more broadly presented as a bioinformatics case study highlighting the biological considerations and computational challenges posed by many microbial eukaryotic genomes due to their evolutionary divergence such as distant homology to known spliceosome genes and high coding density, which are likely to be missed by statistical prediction models or annotation pipelines calibrated using well-studied model eukaryotic organisms. We not only identified previously undetected splicing activity, but also report evidence suggesting that *Giardia* has evolved highly derived mechanisms of intronic splicing and gene expression, the emergence of which may be a common occurrence across the microbial eukaryotic tree of life from an intron-rich ancestor.

## Introduction

The protozoan parasite *Giardia duodenalis* (syn. *G. lamblia*, *G. intestinalis*) is a major pathogen of humans and animals worldwide as the causative agent of giardiasis, a common diarrhoeal illness. Infection is acquired via the faecal–oral route, whereby *Giardia* cysts shed in the faeces of an infected host are ingested by a susceptible host in contaminated water or food, or through person-to-person contact with an infected individual. The cysts are highly resistant to disinfection efforts, leading to a higher propensity to cause sizeable outbreaks of illness [1, 2]. The *G. duodenalis* genome is approximately 12 Mb, consisting of five chromosomes, and lacks typical eukaryotic mitochondria, retaining only a significantly reduced evolutionary remnant called the mitosome [3, 4]. The coding density of the genome is currently estimated at 81.5 % and contains 4963 annotated genes [4]. Initial evolutionary hypotheses posited that this organism was one of the earliest-branching eukaryotic taxa, lacking typical eukaryotic features such as endosymbiotic organelles and introns that were acquired later and, subsequently, proliferated across the eukaryotic branch of life. Alternatively, it is now more widely accepted that *Giardia*’s unique characteristics are the result of reductive evolutionary processes tied to its transition towards obligate parasitism [3, 4].

Recent efforts to complete the fragmented (high-draft) genome of the *G. duodenalis* assemblage A isolate ‘WB’, originally published in 2007 and widely used as the reference genome for *Giardia* research, have made significant progress towards a complete and accurate set of reference gene models, including integrating eight previously published *cis-*splicing introns into the reference annotations [3–9]. Consensus splice-site and branch point motifs inferred from initial studies allowed subsequent studies to develop regular expression models of *Giardia*’s introns to search for additional candidates. However, this approach only identified three additional introns [10, 11]. Thus, it remains an open question whether the small number of identified introns is attributed to *Giardia*’s genome not containing many introns, or to the methods used thus far to identify introns relying heavily on expected sequence conservation and thus not being sensitive to other splicing motifs and/or mechanisms. In line with the latter hypothesis, bioinformatic and experimental characterization of small nuclear RNAs (snRNAs) in *Giardia* have identified homologous key components of both the major and minor eukaryotic spliceosome responsible for classic canonical mRNA-splicing (GT–AG/CT–AG) and minor canonical AT–AC splicing [10]. Further, five *trans*-splicing events were experimentally characterized [11–15], demonstrating that more complex mechanisms of gene expression and regulation are active in *Giardia*. Identifying *Giardia*’s complete intron complement is therefore key to completing the gene annotation of the reference genome and for generating new hypotheses regarding genes and expression mechanisms underlying important traits and epidemiology.

In this work, we gathered a collection of mRNA sequence data from six independent studies published between 2013 and 2022 [6, 16–21] and used these as the basis of a novel bioinformatics investigation of the gene models currently annotated in the *G. duodenalis* reference genome. Thus, our objectives in this study were to identify and characterize novel splicing activity to improve the gene models used for reference genome annotation. We developed and tuned a computational workflow for *cis*-splicing intron detection using available RNA-seq data and known introns as positive controls. Our method identified 34 intron candidates and characterized their properties such as potential to form stable secondary structures, sequence motifs and estimated splicing efficiency. Finally, based on results from intron discovery, we suggest that *Giardia* evolved from intron-rich ancestors, losing large non-essential portions of its genome including elements of its ancient eukaryotic spliceosome, into the current compact genome during its evolutionary transition to an obligate parasite.

## Methods

### Data retrieval

Expressed sequences of *Giardia duodenalis* assemblage A (WB strain) were retrieved from NCBI’s GEO and SRA databases after filtering by type ‘Expression profiling by high throughput sequencing’ to remove hits from microarray expression studies. We excluded microarray data as they do not include raw sequencing reads that can be mapped to a reference genome. We additionally selected only paired-end Illumina short reads sequenced from strand-specific mRNA (cDNA) libraries to maximize the comparability between data generated by different studies and to ensure high mapping accuracy. The most recently updated reference genome for *G. duodenalis* assemblage AI (NCBI accession: GCF_000002435.2 [3, 4] was downloaded from NCBI along with accompanying GTF and transcript/CDS FASTA annotation files.

### Bioinformatic discovery of putative splicing activity

The Nextflow Core (‘nf-core’) pipeline rnaseq v3.6 was used for quality control of raw sequencing data and splice-aware alignment of reads to the reference genome, using the genomic FASTA, transcript FASTA and GTF annotation files as inputs [22, 23]. We modified the output parameters of the rnaseq pipeline’s HISAT2_ALIGN module to include the parameter --novel-splicesite-outfile to detect and report potential novel splice junctions not previously annotated in the reference genomes. The novel splice junction output files for all experimental replicates in the same reference study were combined and dereplicated to generate a list of predicted splice junction (SJ) coordinates. Eight previously experimentally characterized *cis*-splicing introns located in the [2Fe–2S] ferredoxin, *RpL7a*, *Rpn10*, dynein light chain and four hypothetical genes were chosen as positive controls to confirm accurate alignment and detection of splicing activity [3, 6–9, 11]. Since these control loci were characterized using the same laboratory strain of *Giardia* (assemblage A strain WB), we developed and tuned our computational pipeline using only data generated from experiments using the same strain (*n*=70 paired-end sequences).

For each individual sample, evidence of potential splicing activity at each splice junction predicted by HISAT2 was inferred using the following criteria: (i) a minimum of five spliced reads (transreads) spanned the putative exon–exon junction and (ii) read depth coverage dropped by 20 % or greater when crossing an exon–intron boundary relative to the flanking exons (following [24, 25]. A list of SJs meeting these initial criteria was generated by combining SJs for all samples. Since intron lengths are known to exhibit substantial variation and given the dearth of available information on *Giardia*’s intron complement, we chose an arbitrary maximum length of 10 kb for consideration as potential *cis*-splicing introns and marked non-conforming candidate introns as potential *trans*-splicing activity. *Trans-*splicing events occur when two coding exons of the same gene are separated by very large distances within the genome, may be located on the opposite strand or even on separate chromosomes entirely, and comprise five of the previously known 13 splice junctions in *Giardia*. Counts of transreads spanning each SJ were computed using the software regtools v0.5.2 [26]. The number of reference studies that each intron candidate in the master list appeared in was tabulated to determine SJs that appear more ubiquitously across the breadth of compiled experimental conditions. We reasoned that stochastic splicing noise in the raw data was unlikely to appear at the same SJ and meet the above criteria across multiple studies, and therefore final filtering retained only candidate introns that appeared in all independent studies from which data were sourced (=a minimum of four unique transcriptome sequences). Finally, candidate SJs which showed evidence of template switching by reverse transcriptase during cDNA library generation were identified and removed by checking for perfect alignment matches between the candidate intron and the in-frame sequence at the intron boundaries. Candidate SJs which mapped to repeat regions of the reference genome were removed in the same fashion for more conservative results.

### Calculation of splicing efficiency

Bedtools v2.30.0 multicov was used to obtain read depth for each intron candidate’s coordinates, using the -s parameter for strand-specific paired-end data and -split parameter to avoid counting transreads in read counts in this step. The parameters -p and ‘-q 10’ were also employed to only count reads aligned as proper pairs and with mapping quality ≥10. Splicing efficiency was then calculated at each SJ as the average ratio of transreads to read depth at the 5′ position following the method described by Prevorovsky *et al*. [27]. Using this definition, a splicing efficiency of 1.00 indicates that all reads mapped to the 5′ position have been spliced. SJs were marked as ‘often spliced’ if the average efficiency was ≥0.3 (e.g. at least 30 % of the reads were spliced) or ‘rarely spliced’ otherwise.

### Intron secondary structure and identification of splicing motifs

Putative intron sequences were extracted from the WB reference genome and used as input to the program RNAfold (version 2.4.6, part of the ViennaRNA toolkit) to evaluate base-pairing potential to form secondary structures, with the temperature parameter set to 37.0 °C and disallowing base pairing on the first and last 6 nt of the intron sequence, which were assumed to contain a splicing signal [28]. RNAfold outputs a predicted secondary structure model and the associated minimum free energy (MFE) per input sequence. The first and last 15 nt of each potential intron sequence were further extracted and categorized by SJ. Canonical GT–AG and semi-canonical junctions (GC–AG, CT–AG, GT–GG and CT–AT) were considered as major SJs, AT–AC as minor SJs and all others as non-canonical SJs [29]. Major SJs were manually inspected for the presence of potential splicing signals. The R package *ggseqlogo* [30] was additionally used to compute and visualize positional weight matrices for aligned sequences per category.

### Functional categorization of introns

A Perl script was developed to categorize putative introns into mutually exclusive groups based on the predicted splicing site coordinates relative to annotated gene models from RefSeq. Categories were: (i) functional – intron coordinates overlap an existing gene model and removal of the intronic sequence produces a valid protein sequence; (ii) non-functional – removal of the intron sequence from the overlapping gene model produces nonsense codons or results in a frameshift containing internal stop codons; and (iii) undetermined – the intron was unable to be classed into one of the other categories, most often when the splice-site coordinates were located outside any existing gene models. Custom scripts are available from the authors upon request.

### Clustering intron candidates

Five characterizing variables were collected from intron candidates and subjected to dimension reduction using Factor Analysis of Mixed Data (FAMD), a method tuned to handle both numeric and categorical mixed data and project the latent dimensions into a reduced coordinate space [31]. These five dimensions were the intronic sequence length, GC%, minimum free energy, splicing efficiency and SJ category. Hierarchical clustering of Euclidean distances was computed between coordinates of the top two prevailing latent dimensions in order to assign candidate introns to clusters based on shared characteristics identified by FAMD. FAMD and hierarchical clustering were computed using the FAMD() and HCPC() functions respectively in the R package *FactoMineR* [32].

## Results

### Bioinformatic characterization of splicing activity identifies 34 new intron candidates in *Giardia*

**Fig. 1.**
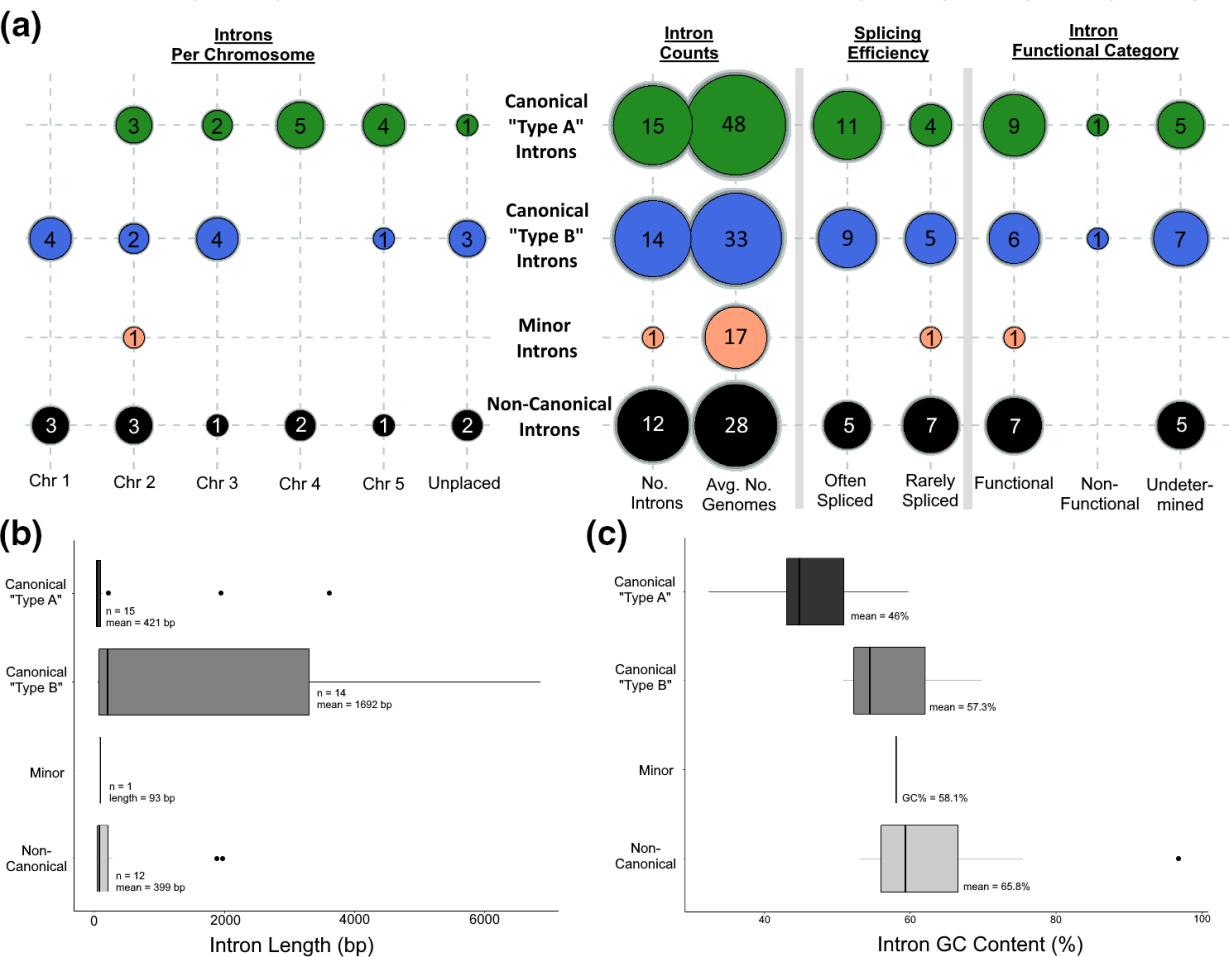
Candidate intron characterizations. Canonical and semi-canonical major introns were separated into two sequence types ‘A’ and ‘B’ based on the first and last 15 bp nucleotides within the intronic sequences. (**a**) Bubble chart of categorical data points per intron class. (**b**) Boxplot of intron lengths. (**c**) Boxplot of intronic sequence GC content. Black bars representing central tendencies in both (**b**) and (**c**) denote the median.

A total of 70 SRA accessions of RNA-seq runs were downloaded from NCBI’s databanks and examined in this study for the presence of splicing activity present in *Giardia*. A summary of samples and their metadata can be found in Table S1, available in the online version of this article. The compiled data were published in six separate studies all using comparable library preparation protocols and Illumina sequencing chemistry [6, 15, 18–21]. From this dataset and our described detection strategy, we identified a total of 42 candidate *cis-*splicing introns, including all eight introns previously characterized using laboratory methods, plus 34 novel ones. The main features of these introns are described in Table 1 and visualized in Fig. 1.

**Table 1.** Characterization of intron candidates in *Giardia duodenalis*

Intron ID	Reference Chromo -some	5' Splice Co -ordinate	3' Splice Coordinate	Strand	Intron Length (bp)	Exon/Intron GC Content	Intron Position	Intron Phase	Exon/Exon Nucleotide Sequence	Splice Junction Nucleotide Sequence (5'/3')	SJ Category	Intron Family	No. (%) Genomes	Splicing Efficiency	MFE (kcal/mol)	Gene ID	Gene Annotation	Spliced Protein Category	Remarks
1	NC_ 051856.1	278 103	278 145	+	42	66.56/ 59.52	4091	2	GTCG/ TTGA	TT-CC	Non-canonical	–	21 (30 %)	Rarely (6.2 %)	−10.0	GL50803_ 0061565	Axoneme- associated protein GASP-180	Functional	
2	NC_ 051856.1	728 909	729 034	–	125	58.26/ 60.00	890	2	CTGA/ GCCG	GC-AG	Major	B	55 (78.6 %)	Rarely (1.6 %)	−33.8	GL50803_ 0017327	Xaa- Pro dipeptidase	Non-functional	
3	NC_ 051856.1	1 090 571	1 090 850	–	279	52.86/ 54.12	1321	1	CTCA/ TGGC	GT-AG	Major	B	26 (37.1 %)	Rarely (4.1 %)	−26.1	GL50803_ 0032778	Ankyrin repeat protein 1	Functional	Possible alternative SJ with intron 4
4	NC_ 051856.1	1 090 571	1 090 664	–	93	53.02/ 53.76	1507	1	CTCA/ TGGC	GT-AG	Major	B	37 (52.9 %)	Rarely (5.2 %)	−93.6	GL50803_ 0032778	Ankyrin repeat protein 1	Functional	Possible alternative SJ with intron 3
5	NC_ 051856.1	1 090 597	1 090 783	+	186	52.85/ 54.84	1574	2	CTCA/ TGCT	TG-CG	Non-canonical	–	18 (25.7 %)	Rarely (1.4 %)	−41.1	GL50803_ 0032778	Ankyrin repeat protein 1	Functional	
6	NC_ 051856.1	1 090 727	1 090 820	–	93	52.96/ 54.84	1351	1	GATA/ TGTG	GT-AG	Major	B	35 (50 %)	Rarely (6.5 %)	−16.8	GL50803_ 0032778	Ankyrin repeat protein 1	Functional	
7	NC_ 051856.1	1 481 251	1 481 283	+	32	74.29/ 96.88	781	1	CGGT/ GCCG	GC-CG	Non-canonical	–	52 (74.3 %)	Often (98.6 %)	−7.6	GL50803_ 00 r40035	18S ribosomal RNA	Undeter -mined	
8	NC_ 051857.1	3862	3894	+	32	74.29/ 96.88	781	1	CGGT/ GCCG	GC-CG	Non-canonical	--	51 (72.9 %)	Often (69.1 %)	−7.6	GL50803_ 00 r40023	18S ribosomal RNA	Undeter -mined	
9	NC_ 051857.1	37 397	37 460	–	63	63.9/ 69.84	923	2	GCAT/ GTAG	GT-AG	Major	B	27 (38.6 %)	Often (47.3 %)	−23.8	GL50803_ 00112304	Elongation factor 1-alpha	Functional	
10	NC_ 051857.1	74 771	74 834	–	63	63.9/ 69.84	923	2	GCAT/ GTAG	GT-AG	Major	B	27 (38.6 %)	Often (53.7 %)	−23.8	GL50803_ 00112312	Elongation factor 1-alpha	Functional	
11	NC_ 051857.1	259 553	259 588	–	35	52.12/ 42.86	22	1	CGCC/ TTAT	CT-AG	Major	A	62 (88.6 %)	Often (76.9 %)	0.0	GL50803_ 0027266	[2Fe-2S] ferredoxin	Functional	Described in Nixon *et al*. 2002 [8]
12	NC_ 051857.1	578 021	578 241	–	220	44.45/ 46.36	90	0	AGGC/ CTCC	GT-AG	Major	A	54 (77.1 %)	Often (61.5 %)	−59.2	GL50803_ 0035332	Un -character -ized protein	Functional	Described in Russell *et al*. 2005 [9]
13	NC_ 051857.1	1 261 544	1 261 581	–	37	50.66/ 32.43	95	2	CCTT/ GAAC	GT-AG	Major	A	53 (75.7 %)	Rarely (1.9 %)	0.0	GL50803_ 0013268	Ribosomal protein S25	Non- functional	
14	NC_ 051857.1	1 505 524	1 505 797	+	273	52.59/ 56.04	1433	2	TTTA/ ACAC	AC-TG	Non-canonical	–	28 (40 %)	Rarely (1 %)	−84.4	GL50803_ 0061420	Ankyrin repeat protein 1	Functional	
15	NC_ 051857.1	1 505 984	1 506 077	+	93	52.78/ 59.14	973	1	TTGA/ CGCA	CG-GG	Non-canonical	–	28(40 %)	Rarely (13.4 %)	−17.7	GL50803_ 0061420	Ankyrin repeat protein 1	Functional	
16	NC_ 051857.1	1 506 016	1 506 109	+	93	52.84/ 58.06	941	2	CCAC/ ATGA	AT-AC	Minor	–	17 (24.3 %)	Rarely (0.5 %)	−19.5	GL50803_ 0061420	Ankyrin repeat protein 1	Functional	
17	NC_ 051858.1	391 795	392 651	+	856	–/63.08	–	–	CGAG/ GTAT	GT-AG	Major	B	21 (30 %)	Often (40.2 %)	−328.0	–	–	Undeter -mined	
18	NC_ 051858.1	392 600	392 651	+	51	–/62.75	–	–	CGAG/ GTAT	GT-AG	Major	B	10 (14.3 %)	Often (32.2 %)	−7.3	–	–	Undeter -mined	
19	NC_ 051858.1	579 597	579 706	+	109	57.23/ 57.80	425	2	TGAA/ CTCG	GT-AG	Major	A	64 (91.4 %)	Often (64 %)	−43.6	GL50803_ 0017244	Ribosomal protein L7a	Functional	Described in Russell *et al*. 2005 [9]
20	NC_ 051858.1	664 300	664 360	+	60	49.67/ 58.33	1005	0	CGCA/ TTGC	TT-TG	Non-canonical	–	55 (78.6 %)	Rarely (25.7 %)	−17.9	GL50803_ 0012148	NEK Kinase	Functional	
21	NC_ 051858.1	1 030 060	1 030 129	+	69	60.22/ 55.07	619	1	ATGA/ GTGC	GT-GG	Major	B	19 (27.1 %)	Rarely (8.7 %)	−9.3	GL50803_ 0010311	Ornithine carbamoyl -transferase	Functional	
22	NC_ 051858.1	1 078 823	1 078 856	–	33	49.5/ 45.45	32	2	TAAC/ GTTC	GT-AG	Major	A	59 (84.3 %)	Often (42.1 %)	−2.5	GL50803_ 0015525	Un -character -ized protein	Functional	Described in Kamikawa *et al*. 2014 [7]
23	NC_ 051858.1	1 855 876	1 859 749	+	3873	–/52.34	–	–	GAAG/ GTGA	GT-AG	Major	B	57 (81.4 %)	Often (61.1 %)	−1383.9	–	–	Undeter -mined	
24	NC_ 051859.1	1 340 429	1 340 465	–	36	44.53/ 36.11	129	0	TAAT/ AGCA	GT-AG	Major	A	57 (81.4 %)	Often (56.1 %)	0.0	GL50803_ 0086945	Un -character ized protein	Functional	Described in Franzen *et al*. 2013 [6]
25	NC_ 051859.1	1 525 530	1 525 587	+	57	58.01/ 75.44	269	2	CAAG/ ATCC	AT-CC	Non-canonical	–	16 (22.9 %)	Rarely (3.1 %)	−15.3	GL50803_ 007878	Ribosomal protein S14	Functional	
26	NC_ 051859.1	1 726 282	1 728 250	+	1968	–/53.15	–	–	TAAT/ AGGA	AG-AC	Non-canonical	–	15 (21.4 %)	Often (74.1 %)	−725.0	–	–	Undeter -mined	
27	NC_ 051859.1	1 878 453	1 878 485	–	32	48.34/ 43.75	3	0	GGCT/ GTCT	GT-AG	Major	A	64 (91.4 %)	Often (67.4 %)	0.0	GL50803_ 0015124	Dynein light chain	Functional	Described in Morrison *et al*. 2007 [3]
28	NC_ 051859.1	2 099 731	2 099 760	+	29	48.61/ 44.83	22	1	ATGT/ CATT	GT-AG	Major	A	62 (88.6 %)	Often (70.1 %)	0.0	GL50803_ 0015604	26S proteasome regulatory subunit Rpn10	Functional	Described in Roy *et al*. 2012 [11]
29	NC_ 051859.1	2 187 227	2 187 309	–	82	–/59.76	–	–	ATGG/ CCAA	GT-AG	Major	A	61 (87.1 %)	Often (91.3 %)	−23.3	–	–	Undeter -mined	
30	NC_ 051859.1	2 585 525	2 587 475	+	1950	–/50.56	–	–	ACAT/ GTCT	GT-AG	Major	A	36 (51.4 %)	Often (60.2 %)	−553.7	–	–	Undeter -mined	
31	NC_ 051860.1	783 046	788 608	+	5562	–/50.83	–	–	TAAG/ GTAC	GT-AG	Major	B	32 (45.7 %)	Often (60.9 %)	−1971.4	–	–	Undeter -mined	
32	NC_ 051860.1	881 711	881 741	+	30	–/43.33	–	–	TCCA/ TATC	CT-AT	Major	A	5 (7.1 %)	Rarely (12.1 %)	0.0	–	–	Undeter -mined	
33	NC_ 051860.1	1 002 717	1 002 758	+	41	48.84/ 51.22	4	1	TGGC/ AAAG	GT-AG	Major	A	62 (88.6 %)	Often (61.6 %)	−1.3	GL50803_ 0060002	Sec61beta family protein	Functional	Described in Kamikawa *et al*. 2014 [7]
34	NC_ 051860.1	1 280 297	1 280 329	+	32	–/43.75	–	–	TACA/ GTTT	GT-AG	Major	A	14 (20 %)	Rarely (2.2 %)	0.0	–	–	Undeter -mined	
35	NC_ 051860.1	1 830 580	1 830 622	+	42	66.56/ 59.52	4091	2	GTCG/ TTGA	TT-CC	Non-canonical	--	19 (27.1 %)	Rarely (4.3 %)	−10.0	GL50803_ 00137716	Axoneme-associated protein GASP-180	Functional	
36	NC_ 051860.1	3 992 739	3 992 769	+	30	48.04/ 40.00	913	1	TAGA/ CTAT	GT-AT	Major	A	23 (32.9 %)	Rarely (1.3 %)	0.0	GL50803_ 0017227	DinF protein	Functional	
37	NW_ 024037079.1	9135	15 988	+	6853	–/52.28	–	–	TAAG/ AAGC	GT-AG	Major	B	26 (37.1 %)	Often (63.6 %)	−2458.2	–	–	Undeter -mined	
38	NW_ 024037084.1	3236	6995	+	3759	–/51.69	–	–	GAGG/ GTGA	GT-AG	Major	B	44 (62.9 %)	Often (96.3 %)	−1326.6	–	–	Undeter -mined	
39	NW_ 024037084.1	5052	6995	+	1943	–/51.42	–	–	GAGG/ GTGA	GT-AG	Major	B	45 (64.3 %)	Often (92.2 %)	−656.8	–	–	Undeter -mined	
40	NW_ 024037099.1	3365	5252	+	1887	–/55.80	–	–	CAGA/ ATCA	AT-TG	Non-canonical	–	19 (27.1 %)	Often (64.8 %)	−689.0	–	–	Undeter -mined	
41	NW_ 024037103.1	5493	5611	+	118	–/63.56	–	–	ACCA/ AGGA	AG-CC	Non-canonical	–	16 (22.9 %)	Often (54.2 %)	−30.9	–	–	Undeter -mined	
42	NW_ 024037104.1	7584	11 200	+	3616	–/51.94	–	–	TTGG/ GTAG	GT-AG	Major	A	44 (62.9 %)	Often (63.2 %)	−1321.2	–	–	Undeter -mined	

Four categories of intron candidates were identified among the 42 candidates based on SJ plus the first and last 15 nt within the intron sequence, which contain additional sequence motifs related to binding sites for the spliceosome as is typical of most known eukaryotic introns. Twenty-nine of the identified introns were classed as canonical or semi-canonical major SJs, 12 were non-canonical SJs, and one SJ was classed as a minor (AT–AC) junction (Fig. 1a). The major SJs could be further sub-classified into two groups, ad hoc named ‘Type A’ and ‘Type B’ introns, based on semi-conserved motifs observed in their internal sequences (Fig. S1). Each of the Type A, Type B and non-canonical introns were identified consistently in at least 40 % (28/70) of transcriptomes, with Type A major introns identified the most consistently in an average of 48 transcriptomes (68.5 %) and Type B major introns in an average of 33 transcriptomes. The sole minor SJ intron was identified in 17 transcriptomes. Average splicing efficiency, the proportion of spliced vs total reads unambiguously mapped to a particular junction, was variable, with a slim majority categorized as often spliced (*n*=25 introns; mean efficiency=0.649; efficiency range=0.322–0.986) and 17 introns categorized as rarely spliced (mean efficiency=0.058; range=0.005–0.257). Each of the three intron categories with more than one representative appear to be evenly dispersed across *Giardia*’s five chromosomes, with Chromosome 2 containing the most introns (*n*=9) and Chromosome 5 containing the fewest (*n*=6). An additional six intron candidates were identified on contigs currently unplaced in the WB reference assembly. In total, 27 introns from all SJ categories were detected within annotated genes, of which 23 produced functional amino acid sequences when the intron was removed by splicing (Fig. 1a). Two introns (one Type A and one Type B) produced non-functional protein sequences when spliced, and an additional two non-canonical introns were identified in separate paralogous copies of the 18S small subunit ribosomal gene. A further 15 introns (five Type A, seven Type B, three non-canonical) did not overlap any known genes. Of the introns which overlap genes, Type A introns are most often found nearest the 5′ start codon of the gene’s reading frame (mean distance from start coding=173 bp/median distance=61 bp), while Type B and non-canonical introns are typically located an average of 1076 and 1666 bp from the start codon (median distances=923 and 1005 bp), respectively (Table 1). Most introns, regardless of category, are short in length – the median lengths of Type A, Type B and non-canonical introns was 36 bp (mean=421 bp), 202 bp (mean=1692 bp) and 77 bp (mean=399 bp) respectively. In addition, the length and GC content (%) distributions further characterized Type A and Type B introns as distinct from one another – the median length of Type B introns is approximately six times greater and they contain GC% content approximately 11 % greater than Type A introns (Fig. 1b, c). Non-canonical introns were shown to be the most GC-rich, with an average GC content of 65.8 % (median=59.4 %; Fig. 1c).

**Fig. 2.**
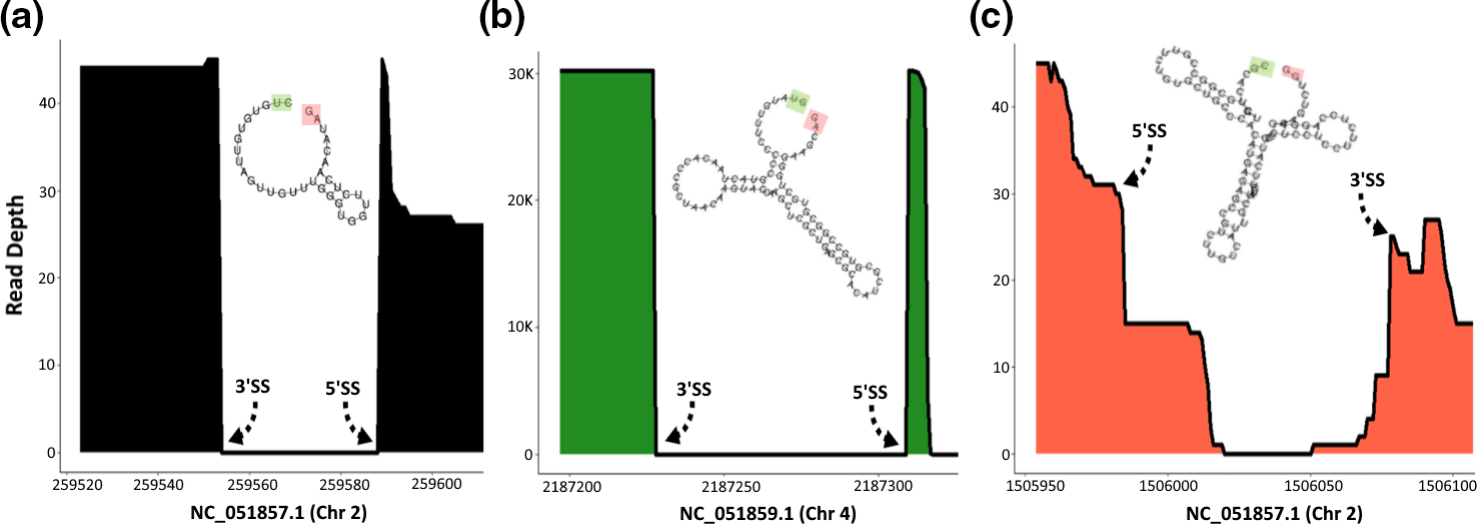
Splice-aware read mapping profiles. The *x*-axis shows chromosomal coordinates in the *G. duodenalis* reference genome. The *y*-axis shows mapped read depth of exonic regions flanking intronic splice site coordinates. Example profiles shown are (a) positive control intron (ID#11 in Table 1), (**b**) representative ‘Type A’, often-spliced intron (ID#29) and (c) representative non-canonical, rarely-spliced intron (ID#15). Predicted RNA secondary structure models are paired with each profile, showing the 5′ splice site highlighted in green and the 3′ splice site highlighted in red. See Table 1 for additional characterization details such as MFE, annotation, etc.

We estimated the ability of each intronic sequence to form stable secondary structures with the program RNAfold, which computes the most probable secondary structure and the associated MFE using a dynamic programming algorithm. Again, the results revealed a clear distinction between Type A and Type B introns, the latter of which form more stable shapes and have an average MFE=−597.1 kcal mol^−1^ (median MFE=−63.7 kcal mol^−1^), while Type A introns have an average MFE=−133.7 kcal mol^−1^ (median MFE=0.0 kcal mol^−1^). Non-canonical introns were overall more similar to Type A introns and had an average MFE=−138.0 kcal mol^−1^ (median=−17.8 kcal mol^−1^). For these measurements, RNA secondary structure can be inferred as less stable or less likely to form any 2D/3D structures as the MFE value approaches 0 kcal mol^−1^. Individual MFE estimates for each intron candidate are reported in Table 1. Selected examples of splice-aware read mapping profiles and RNA secondary structures are shown in Fig. 2.

### Clustering of intron candidates suggests secondary structure plays a greater role in splicing than sequence motifs

**Fig. 3.**
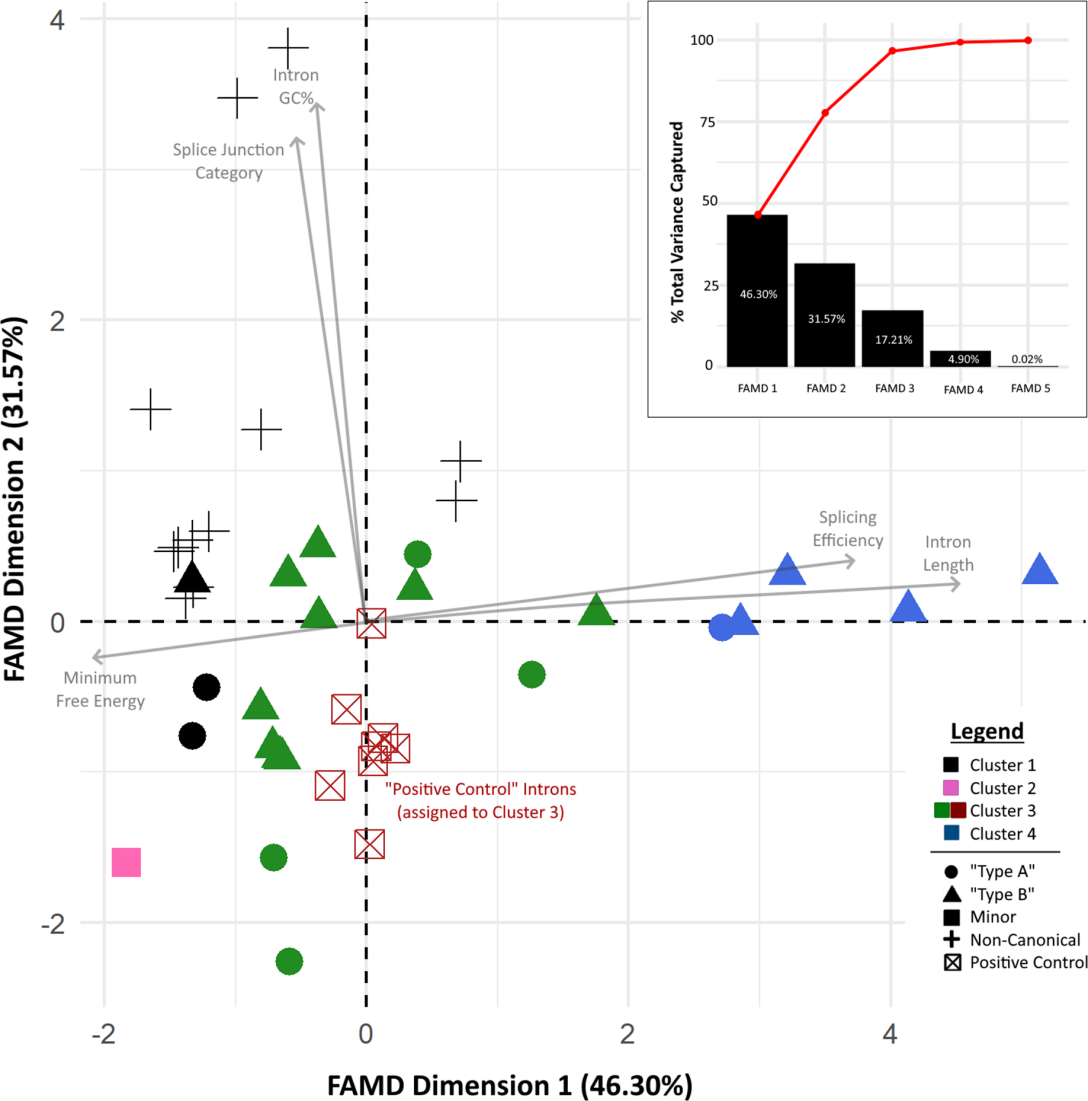
Scatterplot of top two FAMD latent dimensions. Each point represents an intron candidate, with shape/colour coded to reflect its assigned hierarchical cluster assignment. Introns which were previously characterized/known are shown in red boxes with an X and are all assigned to Cluster 3. Inset: scree plot of FAMD dimensions. Black bars represent the amount of total variance amongst data points captured by each latent dimension. Red line represents the cumulative total variance.

FAMD dimension reduction identified four clusters. The first latent dimension (Fig. 3, *x*-axis) strongly correlated with intron length, MFE and average splicing efficiency (*r* ^2^=0.954, –0.953 and 0.617, respectively). The second dimension showed comparably strong correlation to GC% and the SJ category (*r* ^2^=0.890 and 0.833 respectively). Average splicing efficiency was the only factor with good correlation to the third latent dimension (*r* ^2^=0.718). In total, these three dimensions accounted for 95.1 % of the total variability among intron candidates (Fig. 3, inset scree plot). The 12 non-canonical intron candidates were all found in Cluster 1, which also included two Type A introns and one Type B intron (Fig. 3, black data points). The lone Minor category intron appeared in its own cluster (Cluster 2, in pink). All eight positive control introns clustered closely together in Cluster 3 (green and red data points) along with four Type A and nine Type B intron candidates. The remaining four Type B introns and one Type A intron formed Cluster 4 (blue data points).

Visually, the four hierarchical clusters of intron candidates did not reveal high degrees of separation from one another, but rather tended to adhere closely to the two latent dimensional axes, with a majority of data points located near the *x*-axis, nor did they segregate by major/minor/non-canonical category.

### Conserved canonical eukaryotic splicing motif signals are only present in Type A intron candidates

**Fig. 4.**
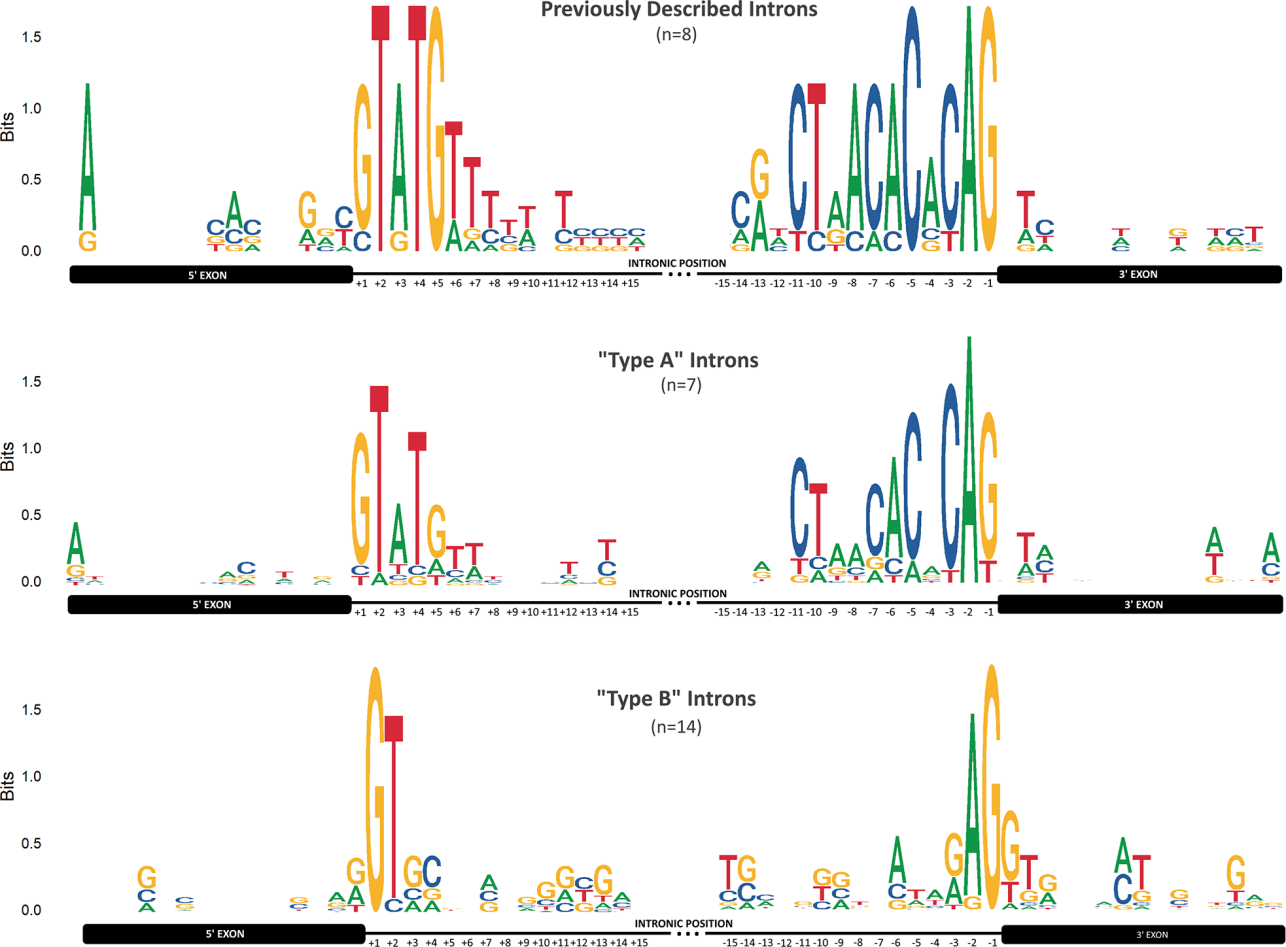
Sequence logos of candidate canonical and semi-canonical ‘major’ introns. Each panel describes the information content (in bits) of the aligned 15 nt in the 5′ and 3′ flanking exonic sequences and, likewise, the 5′ and 3′ 15 nt within the intron sequence. Top and middle panels separate out the sequence logos for the eight ‘positive control’ introns previously characterized in prior studies and the seven newly identified intron candidates classified as ‘A’ type introns, respectively.

The eight positive control introns closely resemble canonical eukaryotic introns, containing the 5′ splicing motif [G|C]TATGT and the fused 3′ splicing and branch point motif CT[A|G]ACACACAG allowing one nucleotide mismatch in the underlined residues [11]. Among the two classes of canonical major intron candidates we examined, we identified similar sequence motifs in the Type A introns, although these motifs were less strongly conserved than those among the positive control introns (Fig. 4, top and centre panels). Conversely, while the Type B introns contain the [G|C]T-AG splice junction motif, none of these introns were observed to contain additional internal sequences resembling the canonical branch point motif upstream of the 3′ splice junction site nor any recognizable 5′ internal motifs (Fig. 4, bottom panel). We visually confirmed this observation by manually aligning and inspecting the extracted intronic sequences (Fig. S1).

## Discussion

This study provides an updated list of proposed intron-containing genes in the *G. duodenalis* genome, expanding the total set of *cis*-splicing introns to 42, including eight experimentally validated introns known from previous studies. We identified a diverse set of intron candidates, most notably among introns containing the canonical major SJs, which can be further categorized into two families based on internal sequence motifs: (i) Type A introns that contain both 5′ and 3′ motifs similar to those recognized by the U2-type spliceosome, and (ii) Type B introns which exhibit little to no sequence motif conservation. Our results provide bioinformatic evidence that *Giardia* has evolved complex, atypical mechanisms of gene expression, potentially due to the reductive processes associated with a transition to obligate parasitism.

Based on its compact genome, early drafts of the *G. duodenalis* genome were *de novo* annotated using prediction models trained on prokaryotic organisms due to the perception that *Giardia* was evolutionarily primitive and contained very few if any spliceosomal introns [3, 8, 9]. Following the confirmation and characterization of the first introns in *Giardia*, subsequent studies used consensus splice-site and branch point motifs in genome-wide *in silico* surveys looking for additional introns but ultimately yielded limited new information. Our study substantially expands the number and type (categorized based on the presumed underlying mechanism) of candidate introns present in the *Giardia* genome compared to previous studies. In addition, *Giardia*’s spliceosome contains a reduced set of proteins and spliceosomal snRNAs understood to be evolutionarily divergent even from its closest genome-sequenced relatives such as *Spironucleus, Trepomonas* and *Kipferlia*. These include secondary structural and motif characteristics of both U2-like/major and U12/minor spliceosomal snRNAs, suggesting that the spliceosome and spliceosomal introns among these organisms are all derived features, each following unique evolutionary trajectories [33–35]. It remains unknown if this reduced, hybrid-like U2/U12 spliceosome displays decreased splicing efficiency in the absence of conserved sequence motifs, pending future experimental exploration of this topic.

### Intronic secondary structure could play an important role in splicing for *Giardia*

Secondary structure-based splicing mechanisms and self-splicing may be common in *Giardia* alongside introns predominantly removed by the spliceosome, a conclusion that has been suggested by other studies that show self-splicing and *trans*-splicing are active in *Giardia* [34–36]. Based on the transcriptomic data used here we observed that Type B and non-canonical introns were often spliced despite not containing the conserved Type A/positive control branch point motif. These introns also tended to have longer lengths and were predicted to form very stable secondary structures throughout the intronic sequence, which may serve a mechanical purpose to bring the two adjacent exons together, similar to our current understanding of *Giardia*’s *trans-*splicing mechanism [7, 15]. This curious property remains in need of further study, as these intron candidates resemble *cis*-splicing introns, but appear to use the splice mechanism suggested for *trans*-splicing events. Alternatively, the reduced spliceosome may also recognize these secondary structures and initiate splicing without requiring sequence motif signals, again more akin to *Giardia*’s *trans*-spliced genes. In this scenario, the involvement of other cofactors or catalyst molecules remains unknown. We also commonly observed rarely spliced Type B and non-canonical introns as generally having low MFE, suggesting that these introns less frequently form stable secondary structures, which are important to *Giardia*’s splicing process. Low MFE is less consequential for Type A introns since these contain conserved branch point motifs recognized by the spliceosome. This secondary structure hypothesis may also explain how introns could persist when two coding genes overlap on opposite strands of the chromosome. That is, sequence motifs as splicing signals would be subject to strong purifying selection as mutations would ultimately affect two coding regions, whereas a secondary structure-based splicing mechanism may reduce selection pressures by allowing an alternative mechanism. From our results, we also cannot rule out the possibility that some introns that form stable secondary structures may also exist in the cell as stable RNAs for example, they may have some additional regulatory functions. Our results here would seem to corroborate the conclusion that *Giardia*’s overall splicing efficiency is variable to low and it is challenging to differentiate between sequencing reads that contain only intronic sequences (i.e. they could be stable RNAs) from those that are part of unspliced transcripts [7].

### The modern *Giardia* genome appears to be derived from an intron-rich ancestor as a consequence of genomic reduction

Evidence that *Giardia*’s few introns are remnants from an intron-rich ancestor can be gleaned from comparisons to genomes of other obligate parasites with free-living relatives in the Fornicata (fornicates) group that includes *Giardia. Spironucleus salmonicida,* a sister species in the family Hexamitidae sharing 70 % small subunit rRNA nucleotide identity with *Giardia,* contains only three known introns [35]. Conversely, *Kipferlia bialata,* a free-living organism considered to be an early branching fornicate lineage, is enriched with introns like most eukaryotic genomes (~125 000 annotated introns [37]). Likewise, *Trichomonas vaginalis* is an obligate parasite related to *K. bialata*, yet it only contains 31 known introns [25]. The patterns of intron loss in *Giardia* thus fit well with existing hypotheses regarding widespread intron loss during the transition to parasitism, a pattern which may still be ongoing within *G. duodenalis* [4, 7]. Interestingly, in contrast to intron loss, we detected an identical 32 bp intronic insertion in two paralogous copies of the nuclear 18S rRNA gene which appears to have remained intact following a gene duplication event. While rare, acquired introns within the 18S rRNA gene have been reported in other microbial eukaryotes and fungi [38–41]. Similarly, two paralogues of the elongation factor 1α gene also each contained an identical 63 bp intron.

While our study focused on short-read RNA-seq of expressed transcripts to identify splice junctions in *Giardia*’s transcriptome, other multi-omics datasets are now available for additional study in the future to validate and polish the SJs we report here and, more broadly, to refine bioinformatics strategies for annotating microbial eukaryote genomes (including intron discovery) [42]. The inclusion of long-read transcriptome sequencing can help further refine the identified SJs due to increased alignment specificity to the flanking exonic regions, and can also be useful for future searches for additional *trans-*splicing junctions [43]. Similarly, MS-based proteome sequencing may also provide additional lines of evidence to support or refute the existence of introns in genes denoted as ‘functional’ in our study (and also *trans*-spliced genes) as well as address the possibility that some introns may also have secondary functions as stable RNAs. This is possible since this type of sequencing captures translated proteins, which would help determine if the seemingly low splicing efficiency we and others have reported is an artefact of mRNA sequencing methods or has greater biological relevance [7, 44]. Further functional confirmation of the novel introns could be determined through the use of CRISPR interference (CRISPRi) methods to knock out genes associated with the spliceosome to repress splicing activity [45, 46].

One aspect of our study that remains unclear is how to consider or contextualize the atypical Type B intron candidates, which exhibit characteristics of both *cis*-splicing and *trans*-splicing mechanisms. Type B introns are comparable in length to, but occasionally longer than, Type A introns but do not contain recognizable branch point motifs. However, Type B introns are definitively characterized by predicted stable secondary structures bringing the disparate exons close to one another as understood for *trans-*splicing based on our analysis. *Trans-*splicing is poorly understood from an evolutionary perspective in microbial eukaryotes but is also recorded from prokaryotes, other protozoan parasites such as *Entamoeba histolytica* and *Trypanosoma cruzi,* and from higher eukaryotes, indicating an early origin of the mechanism [36, 47–49]. *Giardia’*s genome contains four known *trans*-spliced genes (HSP90, p68 RNA helicase, and the dynein heavy chain β and γ elements), which are thought to be nearly universal to all eukaryotes, having evolved very early among the eukaryote ancestral lineage [11–15]. Prior studies examining these *trans*-splicing events in *Giardia* suggest that these genes perhaps existed as single ORFs or ‘typical’ *cis*-spliced multi-exon genes in the ancestral *Giardia* genome, with their coding segments being physically separated by subsequent genome rearrangements across millennia of evolutionary time [14, 15]. It can be hypothesized that Type B introns are an intermediate form between *cis* and *trans-*spliced genes where the intervening rearranged genomic DNA has subsequently been lost, bringing the exons back into close proximity in the genome. Alternatively, these introns may be related to self-splicing Group II introns, which are known from some bacterial and eukaryotic organelles but are not known to exist in any eukaryotic nuclear genome [50–53]. These hypotheses regarding the nature of Type B introns as potential Group II introns remain speculative at present and warrant further experimental study in the future.

## Conclusions

The collective evidence obtained in this study adds additional novel information and context to the intron-rich ancestor hypothesis that *Giardia*’s evolutionary transition from free-living protozoan to obligate parasite involved significant genomic reduction, including loss of many ancient introns and components of the spliceosome required to process them. As a result, we posit that a derived mechanism arose alongside the reductive process towards parasitism, allowing *Giardia* to regulate splicing/gene expression to make use of secondary structure-mediated kinetics to bring exons into close physical proximity and, thus, produce mature mRNAs. This mechanism is perhaps related to, or possibly derived from, the ancient *trans-*splicing mechanism known in *Giardia* and other microbial eukaryotes. However, further genomic comparisons are needed to determine potential origins or homologous mechanisms in related species. Intron candidates identified in this study were characterized by read mapping of experimental RNA-seq data and bioinformatics, and not characterized individually using Sanger-based methods as has been the practice for prior experimental confirmation of intronic splicing in *Giardia*. We acknowledge this difference in characterization methods as a potential limitation of our study. Nonetheless, the RNA data used in our study represented multiple replicates generated from the same isolate of *Giardia* cultured under a variety of experimental conditions that should capture a breadth of stochastic or experimental noise in the raw sequencing data and thus provide high confidence in the results obtained. Future experimental confirmation of these intron candidates will add valuable evidence to this study’s conclusions and aid in the future development/refinement of statistical models of eukaryotic genes in divergent genomes.

## Supplementary Data

Supplementary material 1
