# Supplementary material for: Spliceosomal introns in the diplomonad parasite Giardia duodenalis revisited
Source: Microb Genom. 2023 Nov 7;9(11):001117. doi: 10.1099/mgen.0.001117 (PMC10711314; doi:10.1099/mgen.0.001117)
Supplement: Supplementary material 1 [file mgen-9-1117-s001.pdf]

SUPPLEMENTARY TABLE 1. Summary of RNA-Seq libraries examined						
Sample Information						
SRA No.	Species	Strain	Life Cycle Stage	Condition/Treatment	Library Prep Method	Reference
SRR445165	G. duodenalis WB (AI)	WB-C6 (ATCC 50803)	trophozoite	untreated	TruSeq Stranded mRNA	Franzen et al. 2013 *PLoS Comp Biol*
SRR445166	G. duodenalis WB (AI)	WB-C6 (ATCC 50803)	trophozoite	untreated	TruSeq Stranded mRNA	Franzen et al. 2013 *PLoS Comp Biol*
SRR8589747	G. duodenalis WB (AI)	WB-C6 (ATCC 50803)	trophozoite	G1-S phase	TruSeq Stranded mRNA	Kim et al. 2019 *Korean J Parasitol*
SRR8589748	G. duodenalis WB (AI)	WB-C6 (ATCC 50803)	trophozoite	G1-S phase	TruSeq Stranded mRNA	Kim et al. 2019 *Korean J Parasitol*
SRR8589749	G. duodenalis WB (AI)	WB-C6 (ATCC 50803)	trophozoite	G1-S phase	TruSeq Stranded mRNA	Kim et al. 2019 *Korean J Parasitol*
SRR8589750	G. duodenalis WB (AI)	WB-C6 (ATCC 50803)	trophozoite	G2 phase	TruSeq Stranded mRNA	Kim et al. 2019 *Korean J Parasitol*
SRR8589751	G. duodenalis WB (AI)	WB-C6 (ATCC 50803)	trophozoite	G2 phase	TruSeq Stranded mRNA	Kim et al. 2019 *Korean J Parasitol*
SRR8589752	G. duodenalis WB (AI)	WB-C6 (ATCC 50803)	trophozoite	G2 phase	TruSeq Stranded mRNA	Kim et al. 2019 *Korean J Parasitol*
SRR10919348	G. duodenalis WB (AI)	WB, clone C6 (ATCC 30957)	trophozoite	0h Giardia control	TruSeq Stranded mRNA	Peirasmaki et al. 2020 *Front Gen*
SRR10919350	G. duodenalis WB (AI)	WB, clone C6 (ATCC 30957)	trophozoite	1.5h infection	TruSeq Stranded mRNA	Peirasmaki et al. 2020 *Front Gen*
SRR10919351	G. duodenalis WB (AI)	WB, clone C6 (ATCC 30957)	trophozoite	3h infection	TruSeq Stranded mRNA	Peirasmaki et al. 2020 *Front Gen*
SRR10919352	G. duodenalis WB (AI)	WB, clone C6 (ATCC 30957)	trophozoite	4.5 infection	TruSeq Stranded mRNA	Peirasmaki et al. 2020 *Front Gen*
SRR10919353	G. duodenalis WB (AI)	WB, clone C6 (ATCC 30957)	trophozoite	0h Giardia control	TruSeq Stranded mRNA	Peirasmaki et al. 2020 *Front Gen*
SRR10919355	G. duodenalis WB (AI)	WB, clone C6 (ATCC 30957)	trophozoite	1.5h infection	TruSeq Stranded mRNA	Peirasmaki et al. 2020 *Front Gen*
SRR10919356	G. duodenalis WB (AI)	WB, clone C6 (ATCC 30957)	trophozoite	3h infection	TruSeq Stranded mRNA	Peirasmaki et al. 2020 *Front Gen*
SRR10919357	G. duodenalis WB (AI)	WB, clone C6 (ATCC 30957)	trophozoite	4.5 infection	TruSeq Stranded mRNA	Peirasmaki et al. 2020 *Front Gen*
SRR10919358	G. duodenalis WB (AI)	WB, clone C6 (ATCC 30957)	trophozoite	0h Giardia control	TruSeq Stranded mRNA	Peirasmaki et al. 2020 *Front Gen*
SRR10919360	G. duodenalis WB (AI)	WB, clone C6 (ATCC 30957)	trophozoite	1.5h infection	TruSeq Stranded mRNA	Peirasmaki et al. 2020 *Front Gen*
SRR10919361	G. duodenalis WB (AI)	WB, clone C6 (ATCC 30957)	trophozoite	3h infection	TruSeq Stranded mRNA	Peirasmaki et al. 2020 *Front Gen*
SRR10919362	G. duodenalis WB (AI)	WB, clone C6 (ATCC 30957)	trophozoite	4.5 infection	TruSeq Stranded mRNA	Peirasmaki et al. 2020 *Front Gen*
SRR2642193	G. duodenalis WB (AI)	WB, clone 1B	trophozoite	48hr	TruSeq Stranded mRNA	Ansell et al. 2015 *PLoS Negl Trop Dis*
SRR2642194	G. duodenalis WB (AI)	WB, clone 1B	trophozoite	48hr	TruSeq Stranded mRNA	Ansell et al. 2015 *PLoS Negl Trop Dis*
SRR2642197	G. duodenalis WB (AI)	WB, clone 1B	trophozoite	48hr	TruSeq Stranded mRNA	Ansell et al. 2015 *PLoS Negl Trop Dis*
SRR2642198	G. duodenalis WB (AI)	WB, clone 1B	trophozoite	48hr	TruSeq Stranded mRNA	Ansell et al. 2015 *PLoS Negl Trop Dis*
SRR2642199	G. duodenalis WB (AI)	WB, clone 1B	trophozoite	60hr	TruSeq Stranded mRNA	Ansell et al. 2015 *PLoS Negl Trop Dis*
SRR2642200	G. duodenalis WB (AI)	WB, clone 1B	trophozoite	60hr	TruSeq Stranded mRNA	Ansell et al. 2015 *PLoS Negl Trop Dis*
SRR2642201	G. duodenalis WB (AI)	WB, clone 1B	trophozoite	60hr	TruSeq Stranded mRNA	Ansell et al. 2015 *PLoS Negl Trop Dis*
SRR2642202	G. duodenalis WB (AI)	WB, clone 1B	trophozoite	60hr	TruSeq Stranded mRNA	Ansell et al. 2015 *PLoS Negl Trop Dis*
SRR2642195	G. duodenalis WB (AI)	WB, clone 1B	trophozoite	96hr	TruSeq Stranded mRNA	Ansell et al. 2015 *PLoS Negl Trop Dis*
SRR2642196	G. duodenalis WB (AI)	WB, clone 1B	trophozoite	96hr	TruSeq Stranded mRNA	Ansell et al. 2015 *PLoS Negl Trop Dis*
SRR2642204	G. duodenalis WB (AI)	WB, clone 1B	trophozoite	96hr	TruSeq Stranded mRNA	Ansell et al. 2015 *PLoS Negl Trop Dis*
SRR2642205	G. duodenalis WB (AI)	WB, clone 1B	trophozoite	96hr	TruSeq Stranded mRNA	Ansell et al. 2015 *PLoS Negl Trop Dis*
SRR10368392	G. duodenalis WB (AI)	WB-C6 (ATCC 50803)	trophozoite	untreated	TruSeq Stranded mRNA	Krakovka et al. 2022 *Front Microbiol*
SRR10368393	G. duodenalis WB (AI)	WB-C6 (ATCC 50803)	trophozoite	untreated	TruSeq Stranded mRNA	Krakovka et al. 2022 *Front Microbiol*
SRR10368394	G. duodenalis WB (AI)	WB-C6 (ATCC 50803)	trophozoite	untreated	TruSeq Stranded mRNA	Krakovka et al. 2022 *Front Microbiol*
SRR10368395	G. duodenalis WB (AI)	WB-C6 (ATCC 50803)	trophozoite	untreated	TruSeq Stranded mRNA	Krakovka et al. 2022 *Front Microbiol*
SRR10368396	G. duodenalis WB (AI)	WB-C6 (ATCC 50803)	trophozoite	untreated	TruSeq Stranded mRNA	Krakovka et al. 2022 *Front Microbiol*
SRR10368397	G. duodenalis WB (AI)	WB-C6 M1NR	trophozoite	No-revertant of WB-C6 M1	TruSeq Stranded mRNA	Krakovka et al. 2022 *Front Microbiol*
SRR10368398	G. duodenalis WB (AI)	WB-C6 M1NR	trophozoite	No-revertant of WB-C6 M2	TruSeq Stranded mRNA	Krakovka et al. 2022 *Front Microbiol*
SRR10368399	G. duodenalis WB (AI)	WB-C6 M1NR	trophozoite	No-revertant of WB-C6 M3	TruSeq Stranded mRNA	Krakovka et al. 2022 *Front Microbiol*
SRR10368400	G. duodenalis WB (AI)	WB-C6 M1NR	trophozoite	No-revertant of WB-C6 M4	TruSeq Stranded mRNA	Krakovka et al. 2022 *Front Microbiol*
SRR10368401	G. duodenalis WB (AI)	WB-C6 M1NR	trophozoite	No-revertant of WB-C6 M5	TruSeq Stranded mRNA	Krakovka et al. 2022 *Front Microbiol*
SRR10368402	G. duodenalis WB (AI)	WB-C6 M1	trophozoite	metrionidiazole resistant	TruSeq Stranded mRNA	Krakovka et al. 2022 *Front Microbiol*
SRR10368403	G. duodenalis WB (AI)	WB-C6 M1	trophozoite	metrionidiazole resistant	TruSeq Stranded mRNA	Krakovka et al. 2022 *Front Microbiol*
SRR10368404	G. duodenalis WB (AI)	WB-C6 M1	trophozoite	metrionidiazole resistant	TruSeq Stranded mRNA	Krakovka et al. 2022 *Front Microbiol*
SRR10368405	G. duodenalis WB (AI)	WB-C6 M1	trophozoite	metrionidiazole resistant	TruSeq Stranded mRNA	Krakovka et al. 2022 *Front Microbiol*
SRR10368406	G. duodenalis WB (AI)	WB-C6 M1	trophozoite	metrionidiazole resistant	TruSeq Stranded mRNA	Krakovka et al. 2022 *Front Microbiol*
SRR10368407	G. duodenalis WB (AI)	WB-C6 M2	trophozoite	metrionidiazole resistant	TruSeq Stranded mRNA	Krakovka et al. 2022 *Front Microbiol*
SRR10368408	G. duodenalis WB (AI)	WB-C6 M2	trophozoite	metrionidiazole resistant	TruSeq Stranded mRNA	Krakovka et al. 2022 *Front Microbiol*
SRR10368409	G. duodenalis WB (AI)	WB-C6 M2	trophozoite	metrionidiazole resistant	TruSeq Stranded mRNA	Krakovka et al. 2022 *Front Microbiol*
SRR10368410	G. duodenalis WB (AI)	WB-C6 M2	trophozoite	metrionidiazole resistant	TruSeq Stranded mRNA	Krakovka et al. 2022 *Front Microbiol*
SRR10368411	G. duodenalis WB (AI)	WB-C6 M2	trophozoite	metrionidiazole resistant	TruSeq Stranded mRNA	Krakovka et al. 2022 *Front Microbiol*
SRR10919150	G. duodenalis WB (AI)	WB, clone C6 (ATCC 30957)	encysting trophozoites	0h Giardia control	TruSeq Stranded mRNA	Rojas et al. 2022 *Front Cell Infect Microbiol*
SRR10919151	G. duodenalis WB (AI)	WB, clone C6 (ATCC 30957)	encysting trophozoites	1.5h infection	TruSeq Stranded mRNA	Rojas et al. 2022 *Front Cell Infect Microbiol*
SRR10919152	G. duodenalis WB (AI)	WB, clone C6 (ATCC 30957)	encysting trophozoites	3h infection	TruSeq Stranded mRNA	Rojas et al. 2022 *Front Cell Infect Microbiol*
SRR10919153	G. duodenalis WB (AI)	WB, clone C6 (ATCC 30957)	encysting trophozoites	4.5 infection	TruSeq Stranded mRNA	Rojas et al. 2022 *Front Cell Infect Microbiol*
SRR10919154	G. duodenalis WB (AI)	WB, clone C6 (ATCC 30957)	encysting trophozoites	0h Giardia control	TruSeq Stranded mRNA	Rojas et al. 2022 *Front Cell Infect Microbiol*
SRR10919155	G. duodenalis WB (AI)	WB, clone C6 (ATCC 30957)	encysting trophozoites	1.5h infection	TruSeq Stranded mRNA	Rojas et al. 2022 *Front Cell Infect Microbiol*
SRR10919156	G. duodenalis WB (AI)	WB, clone C6 (ATCC 30957)	encysting trophozoites	3h infection	TruSeq Stranded mRNA	Rojas et al. 2022 *Front Cell Infect Microbiol*
SRR10919157	G. duodenalis WB (AI)	WB, clone C6 (ATCC 30957)	encysting trophozoites	4.5 infection	TruSeq Stranded mRNA	Rojas et al. 2022 *Front Cell Infect Microbiol*
SRR10919159	G. duodenalis WB (AI)	WB, clone C6 (ATCC 30957)	encysting trophozoites	1.5h infection	TruSeq Stranded mRNA	Rojas et al. 2022 *Front Cell Infect Microbiol*
SRR10919160	G. duodenalis WB (AI)	WB, clone C6 (ATCC 30957)	encysting trophozoites	3h infection	TruSeq Stranded mRNA	Rojas et al. 2022 *Front Cell Infect Microbiol*
SRR14252832	G. duodenalis WB (AI)	WB, clone C6 (ATCC 30957)	cyst	1.5h infection	TruSeq Stranded mRNA	Rojas et al. 2022 *Front Cell Infect Microbiol*
SRR14252833	G. duodenalis WB (AI)	WB, clone C6 (ATCC 30957)	cyst	1.5h infection	TruSeq Stranded mRNA	Rojas et al. 2022 *Front Cell Infect Microbiol*
SRR14252834	G. duodenalis WB (AI)	WB, clone C6 (ATCC 30957)	cyst	3h infection	TruSeq Stranded mRNA	Rojas et al. 2022 *Front Cell Infect Microbiol*
SRR14252835	G. duodenalis WB (AI)	WB, clone C6 (ATCC 30957)	cyst	3h infection	TruSeq Stranded mRNA	Rojas et al. 2022 *Front Cell Infect Microbiol*
SRR14252836	G. duodenalis WB (AI)	WB, clone C6 (ATCC 30957)	cyst	3h infection	TruSeq Stranded mRNA	Rojas et al. 2022 *Front Cell Infect Microbiol*
SRR14252837	G. duodenalis WB (AI)	WB, clone C6 (ATCC 30957)	cyst	4.5 infection	TruSeq Stranded mRNA	Rojas et al. 2022 *Front Cell Infect Microbiol*
SRR14252838	G. duodenalis WB (AI)	WB, clone C6 (ATCC 30957)	cyst	4.5 infection	TruSeq Stranded mRNA	Rojas et al. 2022 *Front Cell Infect Microbiol*
SRR14252839	G. duodenalis WB (AI)	WB, clone C6 (ATCC 30957)	cyst	4.5 infection	TruSeq Stranded mRNA	Rojas et al. 2022 *Front Cell Infect Microbiol*

Supplementary Table 1: Summary of RNA-Seq data examined.

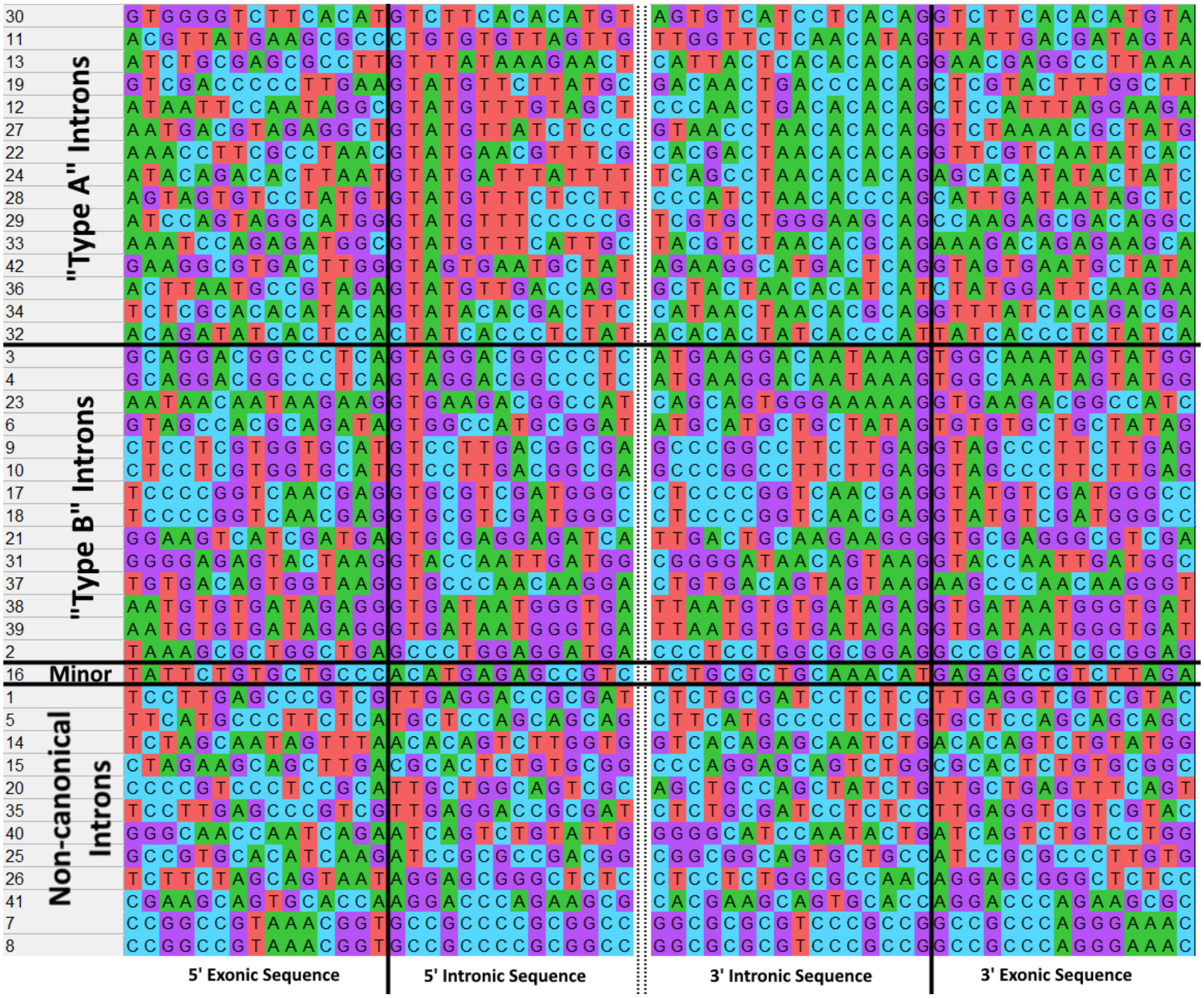

Supplementary Figure 1: Alignment of 42 intronic sequences. The first and last 15 nucleotides belonging to the intron sequence are shown, along with the flanking exonic 15 nucleotides.
